# Pre-Embryonic Period Observation Shows a Unique Reproductive Strategy of the Critically Endangered Anji Salamander (*Hynobius amjiensis*)

**DOI:** 10.3390/ani14203007

**Published:** 2024-10-17

**Authors:** Yu Qiu, Kaiyang Chen, Yiyun Mei, Jia Yang, Cangsong Chen

**Affiliations:** 1 College of Life Sciences, China Jiliang University, Hangzhou 310018, China; qy0125@cjlu.edu.cn; 2 Zhejiang Museum of Natural History, Hangzhou 310014, China; cky123@cjlu.edu.cn (K.C.); meiyy_ok@126.com (Y.M.); yangj@zmnh.com (J.Y.)

**Keywords:** *Hynobius amjiensis*, artificial breeding, embryo development, reproduction strategy, cannibalization

## Abstract

*Hynobius amjiensis*, also known as the Anji salamander, is an amphibian species currently categorized as endangered due to its limited geographical distribution, primarily in China. To address the critical conservation status of this species, artificial breeding is essential for population expansion. However, progress in artificial breeding efforts has been hindered by the scarcity of research on the reproductive biology of the Anji salamander. In this study, we identified 25 distinct early stages of embryo development. Additionally, we observed that Anji salamander embryos contain a lesser amount of yolk compared to other salamanders or frogs. We further discovered that the Anji salamander employs a highly competitive reproductive strategy, producing a smaller number of high-quality offspring. This strategy aims to generate adaptive individuals through intense intraspecific competition, driven by three factors: extremely confined breeding habitats, a substantial number of eggs, and a reduced yolk content. We introduce the term “mass escape” to describe this strategy, which provides a novel perspective on cannibalization, focusing on the consumption of specific body parts rather than a single-individual activity. This study offers valuable insights into artificial breeding techniques designed to mitigate inherent intraspecific competitive pressure, thereby improving metamorphosis and survival rates. Additionally, it provides a comprehensive table detailing the pre-embryonic developmental stages of the Anji salamander.

## Simple Summary

The Anji salamander (*Hynobius amjiensis*) is an endangered amphibian native primarily to China. Artificial breeding is crucial for its population growth, yet research on its reproduction remains limited. This study identified 25 early embryo stages and observed that these embryos contain less yolk compared to those of other salamanders or frogs. The Anji salamander employs a “mass escape” reproductive strategy, producing fewer but higher-quality offspring due to intense competition within its restricted breeding habitats. This research offers new insights into these salamnders’ breeding biology and proposes methods to enhance breeding practices for conservation.

## 1. Introduction

*Hynobius amjiensis*, found exclusively in northern Zhejiang, China, is a member of the Hynobiidae family within the genus *Hynobius*. It maintains a small population in its natural habitat and was classified as Endangered (EN) by the IUCN in 2021. Additionally, it holds a Critically Endangered (CR) status according to the Red List of Biodiversity in China (2021), designating it as a Class I priority wildlife species in China. The Hynobiidae family is believed to have originated no earlier than the Middle Cretaceous period [1]. All members of the Hynobiidae family possess lungs, with the exception of the genus *Onychodactylus* [2]. Species within the genus *Hynobius* are predominantly terrestrial, with juveniles undergoing a brief aquatic phase before transitioning to terrestrial life upon completing metamorphosis within the same year. This is characteristic of their typical metamorphic development. *Hynobius amjiensis* primarily resides in mid-mountain swamps at elevations ranging from 1300 to 1600 m above sea level. Female *Hynobius amjiensis* deposit their eggs in puddles containing moss (mainly sphagnum) and other aquatic vegetation during early winter to early spring, once they have reach sexual maturity. These puddles are characterized by clear water and small volumes, with a breeding pool area ranging from 0.2 to 1.2 square meters and a water depth of 0.7 to 1.2 m [3]. Following egg deposition, females depart from the puddles, while mating males remain to encircle the egg capsules, facilitating in vitro fertilization. Typically, females lay a single pair of egg capsules during a breeding season, each containing approximately 100–150 fertilized eggs. During the peak season, the puddles may contain over 2000 tadpoles. Due to confined breeding habitats, limited food availability, and substantial populations, intense competition for space and resources arises among similar species. Consequently, only approximately 1–3% of subadults successfully complete metamorphosis and reach the shore [4]. This process of selecting dominant individuals is a critical factor contributing to the species’ vulnerability at this stage.

Amphibians, occupying an intermediate evolutionary niche, exhibit a diverse life cycle characterized by distinct stages. This life cycle includes the transformation from free-living aquatic larvae to terrestrial adults, and the maturation of fertilized eggs into subadults that eventually hatch and become terrestrial adults [5]. Initially, the most primitive phase represents the starting stage, with each subsequent stage occurring in different developmental environments, encompassing both aquatic and terrestrial habitats. This bidirectional life cycle has undergone numerous modifications throughout amphibian evolution, resulting in a variety of growth and developmental forms, including non-aquatic larvae. Changes in the life cycle often correlate with varying selection pressures experienced by individuals within diverse ecological contexts [6,7]. Importantly, these modifications do not always require significant genetic-level changes.

When confronted with extreme or unpredictable environmental conditions, which such as temperature fluctuations, changes in water availability, and other selective pressures, amphibian species have evolved a range of reproductive strategies. Among extant amphibian orders, a diverse array of strategies has developed, including the selection of breeding sites, internal and external fertilization, oviparity and viviparity, and various forms of parental care [5,8]. Achieving an optimal balance between egg size and quantity is crucial for successful breeding [9]. Existing research suggests that trade-offs related to egg characteristics are frequently influenced by factors such as environmental conditions, food availability, breeding season duration, and reproductive tactics [10,11,12,13,14]. These factors result in a negative association between egg size and the number of eggs laid [14]. Typically, species increase egg size at the cost of reducing the number of eggs, thereby enhancing the environmental adaptability of their offspring [11,13]. Remarkably, *Hynobius amjiensis* adopts a contrary strategy. It has managed to sustain a significant number of viable populations despite millions of years of environmental changes and degradation [15]. Notable aspects of its reproductive strategy include enduring harsh winter conditions, utilizing confined breeding sites with limited food resources, and producing a large number of offspring [3]. It is reasonable to expect that heightened intraspecific competition would lead to cannibalism. However, this mutual cannibalism appears to be a deliberate and selective process. Research by Fu et al. supports the persistence of cannibalism [16]. A similar phenomenon, referred to as mutual cannibalism in utero, is observed during the embryonic development of fire salamanders [17]. Numerous studies have shown that mutual feeding accelerates larval development, enhances predation capabilities, and improves environmental adaptability [18,19]. The fire salamander, with its unique oviparous strategy among amphibians, serves as a model organism in research examining the transition from oviparity to viviparity. Extensive research has explored its embryonic development and intrauterine mutual feeding [20,21,22]. Recent years have seen limited investigation into the trade-off between egg quantity and size concerning embryonic development. Consequently, this study aimed to elucidate the connection between these factors by examining embryonic development. Only a handful of species within the genus *Hynobius* have been the subjects of pertinent reports on embryonic development [23,24], and there is a notable lack of research on the embryonic development and reproductive strategy of *Hynobius amjiensis*. Thus, a comprehensive understanding of the embryonic development of Anji salamander will improve our knowledge of the reproductive strategy of *Hynobius amjiensis*, enhancing and expanding upon prior research. Additionally, this study explored the external developmental timing and attributes, providing valuable comparative data to extend our understanding of the evolution of amphibian reproductive strategies.

## 2. Materials and Methods

### 2.1. Sample Collection

The breeding season of *Hynobius amjiensis* extends from early December to late April of the following year. We selected the breeding site of *Hynobius amjiensis* in Longchi, Qingliangfeng, Lin’an, Hangzhou, Zhejiang Province, for sample collection at the beginning of the breeding season. A fresh pair of fertilized egg sacs was collected from the breeding pit area on the southeast side of Longchi (30°6′48″ N, 118°52′26″ E; elevation 1609 m above sea level), and we preserved them in a storage container filled with water from the egg sac breeding pit. The container was kept at room temperature, and we transported the samples back to the laboratory on the same day of collection.

### 2.2. Laboratory Observation

In the laboratory, a 200 mm × 200 mm × 150 mm glass container was prepared for sample storage. The fertilized egg sacs were examined under a Carl Zeiss Microscopy GmbH microscope (from Jena, Germany) to ensure their health and successful fertilization, using the associated ZEN 3.0 (blue edition) software for data measurement. Regular monitoring of embryonic development was conducted under the microscope, with observations and recordings produced accordingly. The incubation temperature for the embryos was maintained at 10 ± 1 °C.

## 3. Results

We have comprehensively documented the developmental process of normally fertilized eggs. Our developmental staging table spans from the initial fertilization stage to the eventual hatching and rupture of membranes in *Hynobius amjiensis*. This compilation integrates data from *Hynobius guabangshanensis* [24] with developmental milestones identified in *Hemidactylium scutatum* [25], *Andrias davidianus* [26], *Xenopus laevis* [27,28], and *Ambystoma mexicanum* [29]. We have delineated and described a total of 26 developmental stages, as detailed in Table 1.

### 3.1. Cleavage of the Ovum Stage (Stages 1–7; Figure 1)

**Figure 1:**
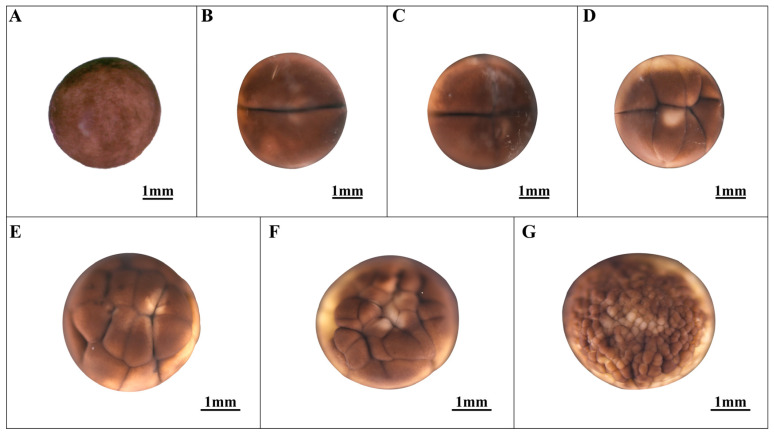
Cleavage of the ovum stage in embryonic development. (**A**) Fertilized egg; (**B**) 2-cell stage; (**C**) 4-cell stage; (**D**) 8-cell stage; (**E**) 16-cell stage; (**F**) 32-cell stage; (**G**) Multi-cell stage.

During fertilization, the egg grains exhibited a suborbicular shape with a diameter of 2.4 ± 0.2 mm. They were enveloped by two distinct membranes: the colloid membrane and the yolk membrane. Both membranes had a gray–black coloration, with no discernible color variations between the plant and animal poles. Notably, the colloid membrane featured an enrichment of colloidal fluid situated between it and the yolk membrane, making it a site of heightened activity within the fertilized egg (referred to as stage 1, the fertilized egg period; see Figure 1A).

The initial cleavage, which occurred meridionally, displayed a symmetrical division pattern, with a furrow extending from the animal pole to both sides of the plant pole, resulting in the formation of two cleavage spheres (designated as stage 2, the 2-cell stage; refer to Figure 1B). Subsequent cleavage events, both meridional and latitudinal, mirrored the cleavage process observed in *Hynobius guabangshanensis*, a species within the same genus [24]. The transition to unequal division characteristics began from the third cleavage (designated as stage 3, the 4-cell stage; refer to Figure 1C; and stage 4, the 8-cell stage; refer to Figure 1D). By the 16-cell stage, division activity became concentrated in the animal pole, resulting in highly irregular cell arrangements (designated as stage 5, the 16-cell stage; refer to Figure 1E). As the cleavage progressed into the multicellular stage of cleavage, cell sizes gradually equalized, and cell boundaries became increasingly indistinct as the cell count increased (designated as stage 7, the multicellular stage; see Figure 1F,G). Consequently, the time duration to reach the multicellular stage was determined to be 146.0 h.

### 3.2. Blastocyst Stage (Stages 8–10; Figure 2)

**Figure 2:**
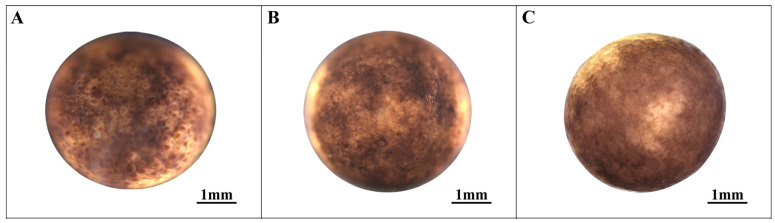
Blastocyst stage in embryonic development. (**A**) Early blastula stage; (**B**) Middle blastula stage; (**C**) Late blastula stage.

As cell division progresses, the embryo advances into the early blastocyst stage. During this phase, the cells on the embryo’s surface become exceptionally diminutive, making it challenging to discern their boundaries. Notably, there is no apparent surface depression at this stage (referred to as stage 8, the early blastocyst; see Figure 2A).

In the middle stage of blastocyst development, the cell boundaries remain indistinct. Surface cells, particularly at the animal pole, tend to aggregate and display irregular cracks and patches, in contrast to the relatively smooth surface observed at the plant pole (designated as stage 9, the middle stage of the blastocyst; see Figure 2B). As the embryo reaches the late blastocyst stage, the cleavage patches at the animal pole dissipate, resulting in a smoother embryo surface (termed stage 10, the late blastocyst stage; see Figure 2B,C). The cumulative duration of embryonic development at this stage is 240.5 h.

### 3.3. Proto-Intestinal Embryo Stage (Stages 11–13; Figure 3)

**Figure 3:**
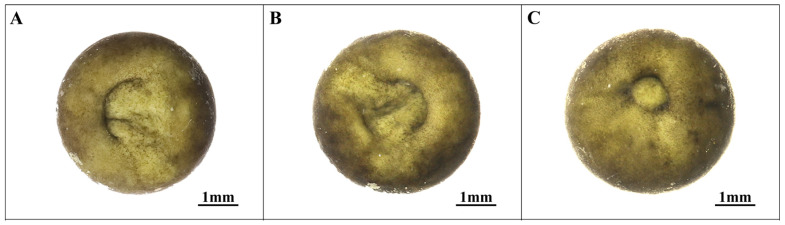
Proto-intestinal embryonic stage in embryonic development of *Hynobius amjiensis*. (**A**) Early gastrula stage; (**B**) Middle gastrula stage; (**C**) Late gastrula stage.

Following the blastocyst stage, embryonic development progresses into the proto-intestinal embryo stage, marking the initiation of formation processes. This phase is characterized by the appearance of a groove at the plant pole, which gradually extends to form a dorsal lip. Ectodermal cells beneath the dorsal lip exhibit inward movement from the vegetative pole and are notably divided into two parts (see Figure 3A).

As the proto-intestine develops, the annulation of the dorsal lip involves more than just unilateral extension: a short concave groove emerges on the opposite side of the dorsal lip (see Figure 3B). These two grooves subsequently extend and merge to create a yolk plug, which gradually reduces in size. The developmental pattern of the proto-intestine in *Hynobius amjiensis* is distinctive, differing from the cell involvement and yolk plug formation patterns observed in most caecilians and anurans, including *Hynobius guabangshanensis*, *Tylototriton shanjing*, *Ambystoma mexicanum*, and *Xenopus* [24,28,30,31,32,33,34,35,36,37,38].

### 3.4. Neural Embryonic Stage (Stages 14–18; Figure 4)

**Figure 4:**
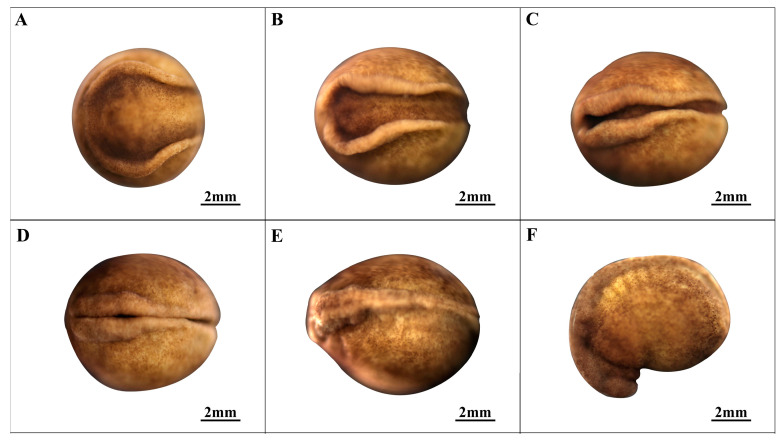
Neural embryonic stage in embryonic development. The embryonic pore is caudal (tail formation) on one side and apical (head formation) on the other. (**A**) Neural plate stage; (**B**) Neural fold stage; (**C**,**D**) Early neural tube stage. (**E**) Middle neural tube stage; (**F**) Late neural tube stage.

In the neural plate stage (stage 14, as shown in Figure 4A), early neural development begins as the proto-intestinal embryo concludes.

**Figure 5:**
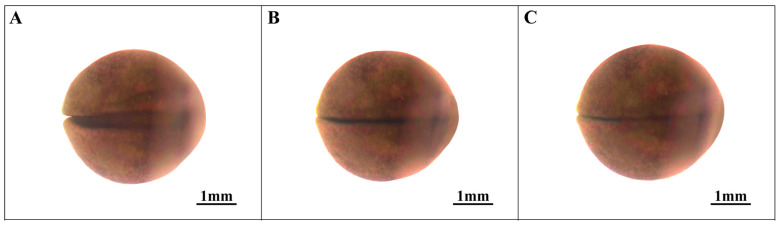
Neural fold line contact patterns in embryonic development. (**A**–**C**) Another medial convergence closure in neural embryonic stage.

This transition involves the gradual narrowing of the blastopore, followed by the invagination and eventual disappearance of the yolk plug. A shallow neural groove becomes evident at the entrance of the foramen ovale, extending from one hemisphere of the embryo to the other (depicted in Figure 4C and Figure 5A). Concurrently, folds form on both sides of the neural sulcus, contributing to the overall extension of the embryo. In the neural fold stage (stage 15, as depicted in Figure 4B), the neural fold bulge thickens and converges toward the center. Concurrently, the outer edge of the apical neural fold widens, while the embryonic opening narrows to a slit. Once the neural fold bulge reaches a certain thickness, it focuses specifically on the neural sulcus, although full closure has not yet occurred. The convergence process exhibits variability, with one form resembling point contact convergence (as shown in Figure 4C,D) and another demonstrating consistent medial convergence closure (depicted in Figure 5A–C).

In the early neural tube stage (stage 16, as seen in Figure 4C,D), the neural fold undergoes gradual closure, resulting in a discernible boundary line. Simultaneously, the embryo extends as the tip and end of the neural fold thicken and close. In the middle neural tube stage (stage 17, illustrated in Figure 4E), the neural crest is essentially closed, with a faintly visible boundary line, and the embryo begins to assume a more grenade-shaped form. During the late neural tube stage (stage 18, as depicted in Figure 4F), the top of the embryo elevates and bulges, giving rise to the head prototype. Simultaneously, somite scars become visible. Additionally, the original embryonic foramen starts to deepen and widen in preparation for the formation of the caudal plate.

### 3.5. Organ Formation Stage (Stages 19–21; Figure 6)

**Figure 6:**
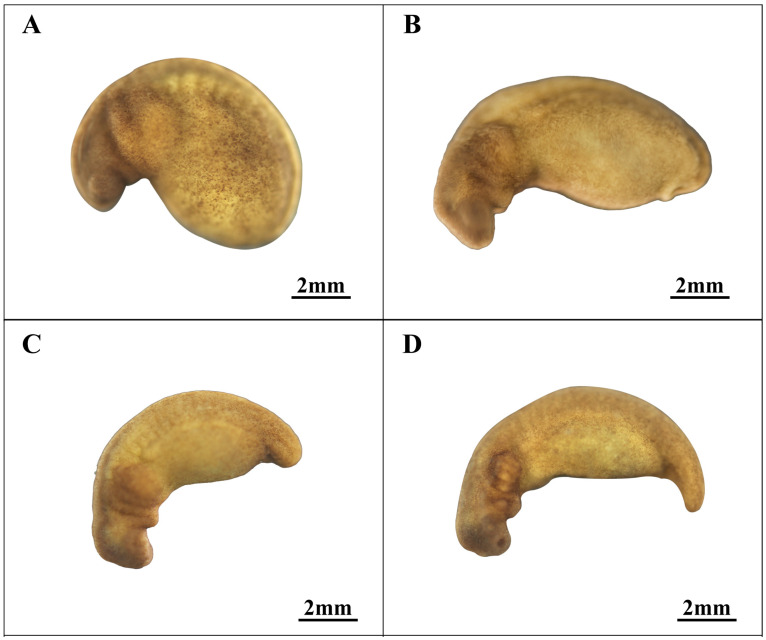
Rapid developmental stage of body length in embryonic development. (**A**) Early tail bud stage; (**B**) Middle tail bud stage; (**C**) Late tail bud stage; (**D**) Early external gill stage.

In the early tailbud stage (stage 19, depicted in Figure 6A), the embryo exhibits a fully formed head shape with prominent, raised eyeballs.

As the yolk content decreases noticeably, the head begins to curve toward the abdomen. Segmental traces of the cheek plate emerge, and the problastodium starts to protrude. In the middle tailbud stage (stage 20, as shown in Figure 6B), there is a significant reduction in yolk content, leading to the overall elongation of the embryo, with growth evident in both the head and tail regions. The head shape reaches its optimal form, with the chin plate differentiating into a chin plate and a balance support plate. The tail bud extends from the embryo’s pore, attaching to the ventral vitelline body. In the terminal caudal bud stage (Stage 21, illustrated in Figure 6C), the head shape continues to evolve, and the anterior segments of the cheek plates on both sides elevate toward the cervical ventral surface, eventually forming the jaw and ventricle. Gradually, the tail buds gain independence as the yolk diminishes, although their distinct shape becomes less pronounced.

During the early stage of external gill development (stage 22, as depicted in Figure 6D), significant changes occur as the embryonic head develops, marking a distinct separation between the head and the trunk. Ventricular development progresses gradually. Gill plates give rise to both outer gills and balance branchial primordia, although these primordia remain connected by a demarcation line. Concurrently, the prominent bulge of the eye vesicles diminishes as they begin to take shape within the eye sockets. The body’s color deepens, and the trunk shows dark, irregular spots. The dorsal region of the body differentiates, while the tail gains independence with rudimentary movement capabilities, though it is not yet fully developed. As the embryo’s body length increases, the space within the yolk membrane becomes insufficient to accommodate its developmental needs. Muscle development intensifies, and the notochord becomes conspicuous, eventually breaking through the yolk membrane due to whole-body twisting.

### 3.6. Pre-Incubation Stage (Stages 22–23; Figure 7 and Figure 8)

**Figure 7:**
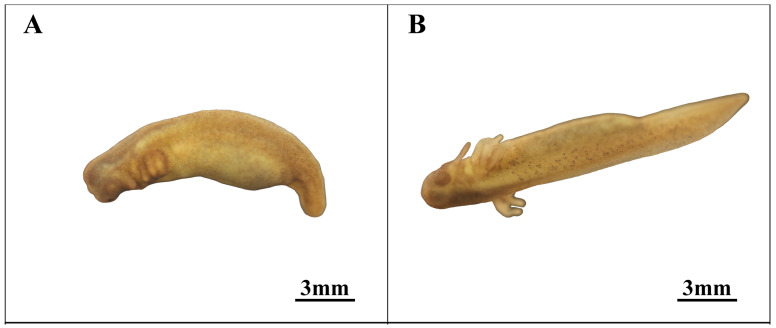
Pre-incubation stage in embryonic development. (**A**,**B**)Middle external gill stage.

Following the rupture of the vitelline membrane during the mid-phase of external gill development (stage 23, depicted in Figure 7A,B), the embryo’s length was 13.3 ± 1.2 mm, and it continued to grow until the embryo fully developed to a body length of 58.3 ± 5.7 mm.

**Figure 8:**
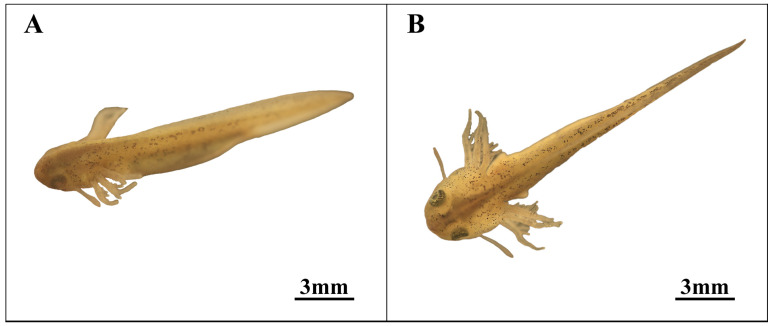
Incubation stage in embryonic development. (**A**) Late external gill stage; (**B**) Incubation stage.

The head progressively takes form, with the anterior portion of the ventral surface initially exhibiting a shallow depression, gradually evolving into the mouth and jaw. The eye color deepens, and the eye socket displays concentric circles, with a lighter center. The demarcation between the head and trunk becomes distinct, while the ventricular prominence gradually diminishes. As the heart develops, internal blood flow and faint pulsations become visible toward the end of this stage. The gills and the balance branch begin to separate, initiating development toward external gills and the balance branch, with external gill development outpacing that of the balance branch. The abdomen gradually diminishes, the yolk further reduces, the caudal division becomes prominent, the cloaca’s location becomes faintly visible, the tail continues to elongate and sharpen, and fins develop on both the upper and lower sides. In the final stage of external gill development (stage 24, as shown in Figure 8A), the external gills grow, and blood flow becomes visible under local microscopic examination.

The eyes have formed but remain dark and unpigmented, with blood flow observable under local magnification. The body length continues to increase, with the tail length approaching and surpassing the carapace length. During this stage, the overall skin transparency increases, the body surface lightens in color, dark spots become more concentrated, and the skin and back muscles develop, so that the notochord becomes visible.

### 3.7. Incubation Stage (Stage 25; Figure 8)

During the incubation stage (Stage 25, as depicted in Figure 8B), the eyeballs become visible, and the external gills reach full development. The body length increases to a specific size, with heightened surface transparency and denser spots. The yolk is depleted, and the distinctive appearance diminishes. Development of the forelimb bud commences, accompanied by frequent membranous activity, indicating imminent membranous hatching. Hatching typically occurs after the forelimb bud emerges, although the exact incubation time remains uncertain.

## 4. Discussion

### 4.1. Yolk and Proto-Intestinal Embryo Development

In comparison to the majority of anuran and caudate species, embryos of the Anji salamander (*Hynobius amjiensis*) exhibit lesser yolk contents (see Table 2).

The reduced yolk levels mean larvae receive insufficient nutrition, which affects post-embryonic development. During the development of *Hynobius amjiensis*, two distinct phases of yolk consumption are observed. The first phase occurs between the late neural tube stage (stage 18) and the gill plate stage (stages 19–20), during which the yolk, initially constituting roughly half of the embryo’s mass, decreases to about one-third. The second phase follows the embryo’s breach of the yolk membrane during outward sensory development. At this point, the yolk’s presence on the embryos is difficult to discern, not becoming conspicuous until the early incubation stage. Once the membrane is ruptured, tadpoles no longer possess sufficient yolk for individual consumption, resulting in undersized trunks. This contrasts with other genera [36,37,38,39]. For instance, in *Echinotriton chinhaiensis*, the yolk proportion during the gill plate stage exceeded half, with this high proportion persisting until the forelimb bud stage, and even after hatching and membrane rupture, the yolk remained visibly present [36]. In most non-mammalian animals, embryonic development takes place within a confined space, relying primarily on yolk proteins stored in yolk granules (Ygs) [40]. In amphibians, Ygs consist mainly of lipoprotein and phosphoprotein structures [41]. Ygs contain modified lysosomal hydrolases that participate in the breakdown of vitellin, supplying nutrients and energy for embryonic development [42,43]. Two distinct mechanisms for yolk degradation have been proposed by researchers. One mechanism proposes the inclusion of hydrolytic enzymes in Ygs during oogenesis, where they remain inactive. Following that, Ygs undergo acidification in the early stages of embryonic development to activate these enzymes [44]. This process has been observed in insects [45,46,47,48], mollusks [42,49], fish [50,51], and amphibians [52,53]. Another significant process entails the introduction of a hydrolytic enzyme required for yolk degradation into Ygs through early lysosome fusion during embryonic development. Subsequently, Ygs fuse with endosomes or lysosomes, activating the enzymes in the process [54]. This process has been observed in the early embryonic growth stages of Japanese salamanders [55] and *brine shrimp* [56], among other organisms.

We tentatively conclude that yolk degradation in *Hynobius amjiensis* aligns with the first method, as the yolk degradation stage corresponds to that observed in *Xenopus laevis*, where yolk consumption initiates at the tail bud stage [54,57].

In comparison to embryos of other anurans, *Hynobius amjiensis* exhibits a yolk content that is significantly less than half of its body volume at the same developmental stages, particularly during the late neural tube stage and early tail bud stage, when observed at a 500 μm scale. In contrast, the yolk contents of *Hyalinobatrachium fleischmanni*, *Hyloxalus vertebralis*, and *Espadarana callistomma* exceed half of their body volume [32,33]. Recent studies have revealed a tissue called the trophic endoderm in fertilized eggs, thought to represent a transitional state in the evolution of amniotic development [58]. In contrast, *Eleutherodactylus coqui* possesses large, fertilized eggs, and within its trophic endoderm, there is a portion of yolk that does not need to supply nutrients to the tissue [30]. This portion of the yolk is likely the primary source of nutrients for the individual during the initial larval stage. It is plausible that *Hynobius amjiensis* embryos are at a more primitive developmental stage and lack a nutritional endoderm, as evidenced by the absence of yolk residue on the surface after hatching from ruptured membranes. This hypothesis requires confirmation through further cytological studies.

The developmental pattern of the proto-intestine in *Hynobius amjiensis* is distinct and specific, characterized by a pattern of cellular engagement and yolk plug formation that sets it apart from species like *Hynobius guabangshanensis* and *Tylototriton shanjing*. In this unique pattern, epithelial motility plays a significant role in reorganization during amphibian proto-intestinal embryogenesis, resulting in a well-defined proto-intestinal anatomy. Experimental observations involving the dissection of proto-intestinal embryos in leopard frogs reveal active movement of mesodermal cells during amphibian proto-intestinal embryo formation. These cells are found to be loosely clustered with significant cell gaps, contrasting with a sticky epithelium [59]. In the development of the proto-intestinal embryo in *Hynobius amjiensis*, cells on the dorsal lip surface display activity, leading to the formation of a brief concave groove on the opposite side of the dorsal lip. This process is followed by the emergence of a distinctive yolk peg pattern as the two grooves extend and merge to create a yolk plug.

**Table 2:** Comparison of the characteristics of some species of the genus *Hynobius* in China.

Species	Body Length (mm)	Egg Diameter (mm)	Number of Eggs/Session	Habitat Altitude (m)
*Hynobius amjiensis*	150.7–180.5	1.6	50–150	1300–1600
*Hynobius formosanus* [60]	58.0–98.0	4.3–5.2	26–32	1800–3650
*Hynobius sonani* [61]	90.0–129.0	5.0	32	2600–3100
*Hynobius leechii* [23]	85.0–142.0	3.0–4.0	56–106	200–850
*Hynobius chinensis* [62]	165.0–205.0	2.4–2.8	66–189	1400–1500
*Hynobius guabangshanensis* [24]	125.0–151.0	2.6–2.8	130–165	720
*Hynobius yiwuensis*	83.9–136.5	2.5–3.1	85–96	100–200

### 4.2. Reproduction Strategy

The yolk quantity is considered to impact egg incubation, hatchling size at hatching, and the environmental adaptability of hatchlings [63,64,65]. The fertilized egg size is intricately linked to the yolk content and influences the asymmetry and asynchrony of early oogenesis, signifying an evolutionary constraint [30,66]. The trade-off between egg size and quantity has been a prominent topic in the examination of species’ reproductive strategies and evolution [14,67,68,69,70,71]. In evolutionary trade-offs, species tend to evolve larger eggs while simultaneously reducing egg numbers, influenced by various selective factors such as larval habitat, direct development, terrestrial spawning, parental rearing forms, etc. [14,67]. Favorable developmental environments have promoted the evolution of larger eggs, resulting in offspring with enhanced environmental adaptations and improved survival [14]. Nevertheless, some species in nature achieve similar goals through an alternative reproductive strategy, laying a multitude of eggs in confined rather than open breeding habitats. While this strategy mitigates the risk of natural predators and interspecific competition to some extent, it subjects the larvae to heightened intraspecific pressure [72,73,74]. Consequently, these species develop specific traits or behaviors in response, such as endogenous nutritional larvae and larvae consuming the same types of eggs [74,75,76,77]. As larval cannibalism is frequently observed in amphibian species under high-pressure conditions, it is typically regarded as a behavior influenced by such environments. During *Hynobius amjiensis* reproduction, a noticeable and self-driven inclination toward increased cannibalism is observed, likely serving to enhance environmental adaptability in subsequent generations.

*Hynobius amjiensis* primarily increases intraspecific pressure through two mechanisms, thereby promoting larval cannibalism within the breeding site. Firstly, similar to the breeding habits of Hylidae and Kalophrynus species, *Hynobius amjiensis* lays its eggs in small pools with volumes not exceeding 2 cubic meters during the winter. This winter egg-laying strategy in small breeding pits helps evade numerous natural predators like snakes and salamanders. However, it also results in limited breeding space and scarce winter food resources. Conversely, when balancing the egg quantity and size, *Hynobius amjiensis* opts for a larger number of smaller fertilized eggs. Field statistics indicate that there are typically 1000–2000 tadpoles in the breeding pit during the breeding season. *Hynobius amjiensis* lays eggs in pairs, typically numbering between 100 and 150 eggs per pair. The actual number can be influenced by factors like food availability, temperature, and maternal health, and it may drop to 50–100. Experiments on embryonic development have shown that *Hynobius amjiensis* follows a consistent strategy concerning egg size and yolk provision. *Hynobius amjiensis* embryos contain just enough yolk to support them until hatching (Figure 8B). After hatching, there is no external or internal nutritional support, which heightens tadpoles’ feeding drive and aggression. In the restricted breeding pit with limited food, conspecific larvae become the primary food source. The energy needed for individuals to undergo metamorphosis is obtained through increased interspecific cannibalism. Simultaneously, the *Hynobius amjiensis* population employs batch spawning. Field data show that each batch’s spawning period lasts over a week, meaning the second batch of eggs is laid just as the first batch is nearing hatching. Laboratory observations reveal that the tadpole larvae of *Hynobius amjiensis* have a hatching closure period of approximately 7 days, partially aligning with the second batch’s hatching time. This leads to differences in body size between the two successive tadpole groups, intensifying intraspecific predation within the breeding pit.

**Figure 9:**
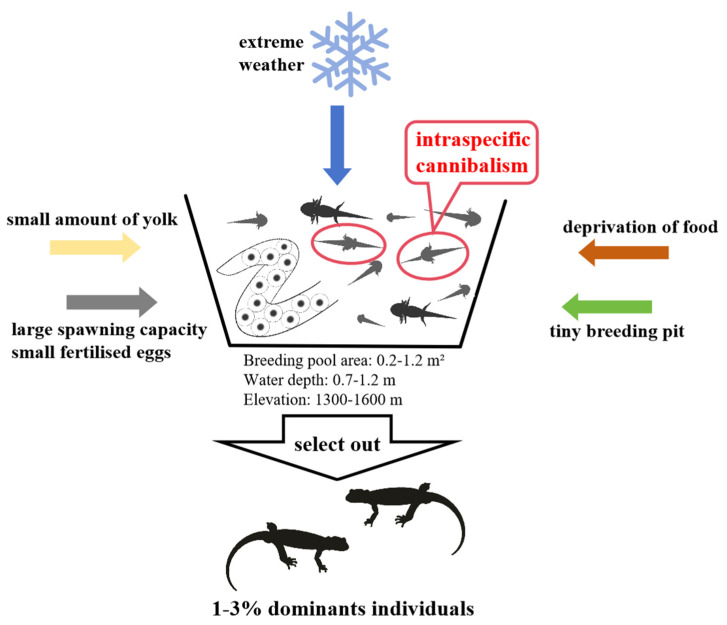
The “mass escape” model.

Based on our experiment observing embryonic development, from the breeding habitat of *Hynobius amjiensis*, to the collection of egg sacs, and to the survival of tadpoles throughout the entire incubation period, along with the study of the yolk content and its consumption during embryonic development, we describe this reproductive strategy as the “mass escape” model (Figure 9).

*Hynobius amjiensis* increases intraspecific tadpole competition and selects individuals with environmental adaptation advantages by creating narrow breeding pits, spawning in winter, producing numerous eggs, and limiting the yolk quantity. Only 1–3% of tadpoles ultimately survive, with over 90% being eliminated during this screening process, serving as a nutrient source for conspecifics. The surviving individuals demonstrate strong environmental adaptability and emerge as the victors in this competition.

This specific reproductive strategy represents a bottleneck in the expansion of the *Hynobius amjiensis* population under current conditions. Enhanced comprehension of *Hynobius amjiensis’* reproductive strategy will facilitate the scientific design of conservation strategies to promote population growth.

## 5. Conclusions

Cao et al. for the first time described in detail the entire developmental staging from spawning to hatching, established a table of normal embryonic development of the Anji salamander, described individual cases of developmental abnormalities, identified and characterized abnormal embryonic amphibians, and filled the information gap in embryonic developmental studies of the small Anji salamander [78]. The present study has extended their work by describing most of the pre-embryonic developmental stages of the Anji salamander, while important phenomena were analyzed in detail and discussed. The complete pre-embryonic developmental stages and characteristics of *Hynobius amjiensis* markedly diverge from those observed in directly developing species [79,80], indicating that *Hynobius amjiensis* follows a more primitive developmental pathway. The early oogenesis of the embryo is holotrophic, akin to that of *Hynobius leechii* [81], *Hynobius chinensis* [62], and *Hynobius guabangshanensis* [24]. Notably, two substantial phases of yolk consumption transpire during pre-embryonic development: stages 18 to 20 and 21 to 23, aligning with significant morphological transformations. Furthermore, it was noted that *Hynobius amjiensis* exhibits a tendency for intraspecific competition during early embryonic development. Understanding this specific reproductive strategy will aid in alleviating intraspecific competition pressure and mitigating cannibalism in captive breeding, ultimately enhancing metamorphosis rates. This study also revealed that there is a tendency for the Anji salamander to exert intraspecific pressure during the early stages of embryonic development. Elucidating this particular reproductive strategy will help us to reduce intraspecific competitive pressure and prevent cannibalism in captive breeding, thereby increasing the metamorphosis rate. Overall, the presented study has produced useful ideas for clarifying the endangerment mechanism of the Anji salamander and developing appropriate conservation strategies and artificial breeding.

## Figures and Tables

**Table 1:** Timetable of embryonic development and staging of *Hynobius amjiensis*.

Number	Developmental Time	Time of Emergence (Since Incubation)/h	Distinguishing Characteristics
1	Fertilized egg (Figure 1A)	0.0	The fertilized eggs are spherical, with a grayish-black color; the animal pole matches the plant pole.
2	2-cell stage (Figure 1B)	8.5	Unequal oogenesis results in the formation of two distinct ovoid spheres.
3	4-cell stage (Figure 1C)	20.0 ± 0.5	Fertilized eggs are 4 cells.
4	8-cell stage (Figure 1D)	34.7	Fertilized eggs are 8 cells.
5	16-cell stage (Figure 1E)	68.4	Fertilized eggs are 16 cells.
6	32-cell stage (Figure 1F)	106.0 ± 0.5	Fertilized eggs are 32 cells.
7	Multi-cell stage (Figure 1G)	146.0	The germ ball undergoes division, resulting in numerous unequal cells.
8	Early blastula stage (Figure 2A)	172.6	Surface cells, although dense and small, remain distinguishable.
9	Middle blastula stage (Figure 2B)	204.3	The fertilized eggs exhibit irregular cracks and patches with unclear cell boundaries.
10	Late blastula stage (Figure 2C)	240.5	The surface gradually smooths from bottom to top, and cell boundaries become indistinguishable.
11	Early gastrula stage (Figure 3A)	252.0	Short, shallow furrows emerge near the equator, and the dorsal sublabial cells initiate invagination.
12	Middle gastrula stage (Figure 3B)	263.4	The dorsal lip extends nearly halfway, and three gradually increasing depressions appear on the opposite side, eventually joining to form a short concave groove.
13	Late gastrula stage (Figure 3C)	266.8	Yolk deposits are present on both sides where the concave groove converges.
14	Neural plate stage (Figure 4A)	274.4	The yolk plug narrows, and the embryo exhibits slight folding with projections on both sides.
15	Neural fold stage (Figure 4B)	300.5	The lateral nerve folds are prominently elevated and converge toward the center.
16	Early neural tube stage (Figure 4C,D)	312.2	The neural folds do not completely fuse, resulting in a spindle-shaped embryo.
17	Middle neural tube stage (Figure 4E)	318.5	The neural crest is nearly closed, and the embryonic shape is well-defined.
18	Late neural tube stage (Figure 4F)	326.8	The rudimentary head, distinct dorsal ridge, and visible segmental marks are apparent.
19	Early tail bud stage (Figure 6A)	345.5	The head is well-formed, with visible eye vesicles and emerging segmented cheek plates.
20	Middle tail bud stage (Figure 6B)	386.0	Both the head and tail elongate, gill plates segment, ventricles develop, and the tail exhibits distinct features.
21	Late tail bud stage (Figure 6C)	428.4	The body length increases, the tail bud diminishes along with the yolk, and the ventricle develops further, resulting in an overall tadpole form.
22	Early external gill stage (Figure 6D)	487.7	The body coloration is distinct, the head, neck, and ventral regions are pronounced, the outer cheek ridge bears visible bud-like structures, no eye sockets are present, and tail characteristics are evident.
23	Middle external gill stage (Figure 7A,B)	551.0	The well-formed head features developed eye sockets and eye structures, there is a visible ventricular heartbeat, and an independent tail separates from the abdomen.
24	Late external gill stage (Figure 8A)	688.7	The eyes take shape, outer gills develop into whisker-like buds, the tail broadens, and the notochord becomes visible.
25	Incubation stage (Figure 8B)	823.4	Development is essentially complete, marked by the emergence of the eye and increased activity within the yolk membrane.
26	Incubation	1054.6	The film is broken during incubation.

## Data Availability

The data supporting the conclusions of this study will be made available from the authors upon request.

## References

[1] Tang D., Xu T.J., Sun Y.N. (2015). Hynobiidae origin in middle Cretaceous corroborated by the new mitochondrial genome of *Hynobius chinensis*. Mar. Genom..

[2] Fei L., Hu S.Q., Ye C.Y. (2006). Amphibia: Gymnophiona, Urodela. Fauna Sinica.

[3] Chen C.S., Yang J., Wu Y.K., Fan Z.Y., Lu W.W., Chen S.H., Yu L.P. (2016). The breeding ecology of a critically endangered salamander, *Hynobius amjiensis* (Caudata: Hynobiidae), endemic to eastern China. Asian Herpetol. Res..

[4] Gu H.Q., Ma X.M., Wang J., Du Z.H., Lou X.Q. (1999). Population size and dynamics of *Hynobius amjiensis*. Sichuan J. Zool..

[5] Duellman W.E., Trueb L. (1986). Biology of Amphibians.

[6] Wake D.B., Roth G. (1989). The linkage between ontogeny and phylogeny in the evolution of complex systems. Complex Organismal Functions: Integration and Evolution in Vertebrates.

[7] Hanken J. (1999). Larvae in Amphibian Development and Evolution. The Origin and Evolution of Larval Forms.

[8] Wells K.D. (2007). The Ecology and Behavior of Amphibians.

[9] Lack D.L. (1947). Data from: Darwin’s Finches: An Essay on the General Biological Theory of Evolution.

[10] Gomez-Mestre I., Pyron R.A., Wiens J.J. (2012). Phylogenetic analyses reveal unexpected patterns in the evolution of reproductive modes. Evolution.

[11] Rollinson N., Hutchings J.A. (2010). Why does egg size increase with maternal size? Effects of egg size and egg density on offspring phenotypes in Atlantic salmon (*Salmo salar*). Evol. Ecol. Res..

[12] Liu Y.J., Zhou S.J., Hu J., Ma Z.H. (2018). Research progress on environmental stress in aquatic animals. J. Tianjin Agric. Univ..

[13] Xu F., Yang W.K., Li Y.M. (2019). Enlarged egg size increases offspring fitness of a frog species on the Zhoushan Archipelago of China. Sci. Rep..

[14] Furness A.I., Venditti C., Capellini I. (2022). Terrestrial reproduction and parental care drive rapid evolution in the trade-off between offspring size and number across amphibians. PLoS Biol..

[15] Yang J., Chen S.H., Chen S.H., Ding P., Fan Z.Y., Lu Y.W., Yu L.P., Lin H.D. (2016). Population genetic structure of critically endangered salamander (*Hynobius amjiensis*) in China: Recommendations for conservation. Genet. Mol. Res..

[16] Fu J.Z., Mark H.H., Liu Z.Z., Zeng X.M. (2003). Genetic divergence of the southeastern Chinese salamanders of the genus *Hynobius*. Acta Zool. Sin..

[17] Weisrock D.W., Papenfuss T.J., Macey J.R., Litvinchuk S.N., Polymeni R., Ugurtas I.H., Larson A. (2006). A molecular assessment of phylogenetic relationships and lineage accumulation rates within the family Salamandridae (Amphibia, Caudata). Mol. Phylogenet. Evol..

[18] Crossland M.R., Shine R. (2010). Cues for cannibalism: Cane toad tadpoles use chemical signals to locate and consume conspecific eggs. Oikos.

[19] Crossland M.R., Shine R., Haramura T. (2023). A biological invasion reduces rates of cannibalism by Japanese toad tadpoles. Sci. Rep..

[20] Dopazo H., Alberch P. (1994). Preliminary results on optional viviparity and intrauterine siblicide in *Salamandra salamandra* populations from northern Spain. Mertensiella.

[21] Greven H. (1998). Survey of the oviduct of salamandrids with special reference to the viviparous species. J. Exp. Zool..

[22] Buckley D., Alcobendas M., Garcia-Paris M., Wake M.H. (2007). Heterochrony, cannibalism, and the evolution of viviparity in *Salamandra salamandra*. Evol. Dev..

[23] Park Y.U., Yoon C.S., Kim J.H., Park J.H., Cheong S.W. (2010). Numerical variations and spontaneous malformations in the early embryos of the Korean salamander, *Hynobius leechii*, in the farmlands of Korea. Environ. Toxicol..

[24] Mi X.Q., Deng X.J., Guo K.J., Niu Y.D., Zhou Y. (2007). Preliminary observations on early embryonic development of *Hynobius guabangshanensis*. Sichuan J. Zool..

[25] Hurney C.A., Babcock S.K., Shook D.R., Pelletier T.M. (2015). Normal table of embryonic development in the four-toed salamander, *Hemidactylium scutatum*. Mech. Dev..

[26] Luo J., Xiao Y., Luo K., Huang X. (2007). Embryonic development and organogenesis of Chinese giant salamander, *Andrias davidianus*. Prog. Nat. Sci..

[27] Nieuwkoop P.D., Faber J. (1994). Normal Table of Xenopus Laevis (Daudin).

[28] Zahn N., James Z.C., Ponferrada V.G., Adams D.S. (2022). Normal table of *Xenopus* development: A new graphical resource. Development.

[29] Bordzilovskaya N.P., Dettlaff T.A., Duhon S.T., Malacinski G.M., Armstrong J.B., Malacinski G.M. (1989). Developmental-stage series of axolotl embryos. Developmental Biology of the Axolotl.

[30] Collazo A., Keller R. (2010). Early development of *Ensatina eschscholtzii*: An amphibian with a large, yolky egg. EvoDevo.

[31] Xiong R.C., Jiang J.P., Fei L., Wang B. (2010). Embryonic development of the concave-eared torrent frog with its significance on taxonomy. Zool. Res..

[32] Hervas F., Torres K., Montenegro P., Pino E.M.D. (2015). Development and gastrulation in *Hyloxalus vertebralis* and *Dendrobates auratus* (Anura: Dendrobatidae). Amphib. Reptile Conse..

[33] Salazar N.M., Del P.E. (2015). Early development of the glass frogs *Hyalinobatrachium fleischmanni* and *Espadarana callistomma* (Anura: Centrolenidae) from cleavage to tadpole hatching. Amphib. Reptile Conse..

[34] Jiang P., Nelson J.D., Leng N., Collins M. (2016). Analysis of embryonic development in the unsequenced axolotl: Waves of transcriptomic upheaval and stability. Dev. Biol..

[35] Zou P.Z., Wen C.Y., Xu J., Chen J.R. (2001). Preliminary study on early embryonic development of *Fejervarya limnocharis*. Chin. J. Zool..

[36] Xie F., Fei L., Li C., Ye C.Y. (2001). Study on early individual development of *Tylototriton zhenhaiensis*. Chin. J. Zool..

[37] Xiang S.J., Deng X.J., Xu J., Xiao Z.L. (2010). Early embryonic development of *Tylototriton wenxianensis*. Chin. J. Zool..

[38] Yang G.H., Yang Z.Z., Li M.R., Wang Z.Q., Song Y. (2011). Preliminary observations on the embryonic development of *Cynops orientalis*. Bull. Biol..

[39] Bernabò I., Brunelli E. (2019). Comparative morphological analysis during larval development of three syntopic newt species (Urodela: Salamandridae). Eur. Zool. J..

[40] Fagotto F., Maxfield F.R. (1995). Changes in yolk platelet pH during *Xenopus laevis* development correlate with yolk utilization. J. Cell Sci..

[41] Ward R.T. (1978). The origin of protein and fatty yolk in *Rana pipiens* IV. Secondary vesicular yolk formation in frog oocytes. Tissue Cell.

[42] Mallya S.K., Partin J.S., Valdizan M.C., Lennarz W.J. (1992). Proteolysis of the major yolk glycoproteins is regulated by acidification of the yolk platelets in sea urchin embryos. J. Cell Biol..

[43] Ramos I., Machado E., Masuda H., Gomes F. (2022). Open questions on the functional biology of the yolk granules during embryo development. Mol. Reprod. Dev..

[44] Fagotto F. (1996). Regulation of yolk degradation, or how to make sleepy lysosomes. J. Cell Sci..

[45] Fagotto F. (1990). Yolk degradation in tick eggs: II. Evidence that cathepsin L-like proteinase is stored as a latent, acid-activable proenzyme. Arch. Insect Biochem. Physiol..

[46] Fausto A.M., Gambellini G., Mazzini M., Cecchettini A., Masetti M., Giorgi F. (2001). Yolk granules are differentially acidified during embryo development in the stick insect *Carausius morosus*. Cell Tissue Res..

[47] Motta L.S., da Silva W.S., Oliveira D.M., de Souza W., Machado E.A. (2004). A new model for proton pumping in animal cells: The role of pyrophosphate. Insect Biochem. Mol. Biol..

[48] Almeida E.D., Dittz U., Pereira J., Walter-Nuno A.B., Paiva-Silva G.O., Lacerda-Abreu M.A., Meyer-Fernandes J.R., Ramos I. (2023). Functional characterization of maternally accumulated hydrolases in the mature oocytes of the vector *Rhodnius prolixus* reveals a new protein phosphatase essential for the activation of the yolk mobilization and embryo development. Front. Physiol..

[49] Schuel H., Wilson W.L., Wilson J.R., Bressler R.S. (1975). Heterogeneous distribution of ‘lysosomal’ hydrolases in yolk platelets isolated from unfertilized sea urchin eggs by zonal centrifugation. Dev. Biol..

[50] Nussenzveig R.H., Oliveira P.L., Masuda H. (1992). Identification of yolk platelet-associated hydrolases in the oocytes of *Rhodnius prolixus*. Arch. Insect Biochem. Physiol..

[51] Busson-Mabillot S. (1984). Endosomes transfer yolk proteins to lysosomes in the vitellogenetic oocyte of the trout. Biol. Cell.

[52] Fagotto F. (1991). Yolk degradation in tick eggs: III. Developmentally regulated acidification of the yolk spheres. Dev. Growth Differ..

[53] Fagotto F., Maxfield F.R. (1994). Yolk platelets in *Xenopus oocytes* maintain an acidic internal pH which may be essential for sodium accumulation. Biol. Cell.

[54] Jorgensen P., Steen J.A.J., Steen H., Kirschner M.W. (2009). The mechanism and pattern of yolk consumption provide insight into embryonic nutrition in *Xenopus*. Development.

[55] Komazaki S., Hiruma T. (1999). Degradation of yolk platelets in the early amphibian embryo is regulated by fusion with late endosomes. Dev. Growth Differ..

[56] Perona R.J.C.B., Vallejo C.G. (1988). Degradation of yolk in the brine shrimp Artemia. Biochemical and morphological studies on the involvement of the lysosomal system. Biol. Cell.

[57] Selman G.G., Pawsey G.J. (1965). The utilization of yolk platelets by tissues of *Xenopus* embryos studied by a safranin staining method. J. Embryol. Exp. Morphol..

[58] Elinson R.P. (2009). Nutritional endoderm: A way to breach the holoblastic-meroblastic barrier in tetrapods. J. Exp. Zool. B Mol. Dev. Evol..

[59] Leblanc J., Yoder M., Brick I. (1988). Morphologic interactions between cells of the anteriad migrating fold during *Rana pipiens* gastrulation. Ann. N. Y. Acad. Sci..

[60] Chang Y.H., Poyarkov N., Vassilieva A., Lai J.S. Reproduction biology, behaviour, and ontogeny in Formosan salamander, *Hynobius formosanus* (Caudata: Hynobiidae), from Taiwan. Proceedings of the 15th European Congress of Her pe tology and SEH Ordinary General Meeting.

[61] Iizuka K., Kezer J., Seto T. (1988). Karyotypes of two rare species of hynobiid salamanders from Taiwan, *Hynobius sonani* (Maki) and *Hynobius formosanus* Maki (Urodela). Genetica.

[62] Piersol W.H. (1910). The habits and larval state of *Plethodon cinereus erythronotus*. Trans. Can. Inst..

[63] Qu Y.F., Zhao S.Z., Jiang X.F., Lin L.H., Ji X. (2019). Can snakes use yolk reserves to maximize body size at hatching?. Curr. Zool..

[64] Allen J.D., Zakas C., Podolsky R.D. (2006). Effects of egg size reduction and larval feeding on juvenile quality for a species with facultative feeding development. J. Exp. Mar. Biol. Ecol..

[65] Landberg T. (2014). Embryonic yolk removal affects a suite of larval salamander life history traits. J. Exp. Zool..

[66] Gould S.J. (1989). A developmental constraint in Cerion, with comments on the definition and interpretation of constraint in evolution. Evolution.

[67] Sargent R.G., Taylor P.T., Gross M.R. (1987). Parental care and the evolution of egg size in fishes. Am. Nat..

[68] Gould J., Beranek C., Valdez J., Mahony M. (2022). Quantity versus quality: A balance between egg and clutch size among Australian amphibians in relation to other life-history variables. Austral Ecol..

[69] Gatto C.R., Robinson N.J., Spotila J.R., Paladino F.V., Tomillo P.S. (2020). Body size constrains maternal investment in a small sea turtle species. Mar. Biol..

[70] Summers K., McKeon C.S., Heying H. (2006). The evolution of parental care and egg size: A comparative analysis in frogs. Proc. R. Soc. B.

[71] Dani K.G.S., Kodandaramaiah U. (2017). Plant and animal reproductive strategies: Lessons from offspring size and number trade-offs. Front. Ecol. Evol..

[72] Lannoo M.J., Townsend D.S., Wassersug R.J. (1987). Larval life in the leaves: Arboreal tadpole types, with special attention to the morphology, ecology, and behavior of the oophagous *Osteopilus brunneus* (Hylidae) larvae. Fieldiana Zool..

[73] Malkmus R., Dehling J.M. (2008). Anuran amphibians of Borneo as phytotelm-breeders—A synopsis. Herpetozoa.

[74] Vassilieva A., Nguyen T. (2023). Restricting living space: Development and larval morphology in sticky frogs (Microhylidae: Kalophrynus) with different reproductive modes. Vertebr. Zool..

[75] Vera Candioti M.F., Nuñez J.J., Úbeda C. (2011). Development of the nidicolous tadpoles of *Eupsophus emiliopugini* (Anura: Cycloramphidae) until metamorphosis, with comments on systematic relationships of the species and its endotrophic developmental mode. Acta Zool..

[76] Formas J.R. (2013). External morphology, chondrocranium, hyobranchial skeleton, and external and internal oral features of *Rhinoderma rufum* (Anura, Rhinodermatidae). Zootaxa.

[77] Cao Z.H., Guo R.Y., Fang Z.Y., Wang Z.W., Liu Y., Lin L.H., Ji X. (2024). Normal table of embryonic development in the Anji salamander *Hynobius amjiensis* (Hynobiidae). Dev. Biol..

[78] Kerney R. (2011). Embryonic staging table for a direct-developing salamander, *Plethodon cinereus* (Plethodontidae). Anat. Rec..

[79] Miller L. (1944). Notes on the eggs and larvae *Aneides lugubris*. Copeia.

[80] Jiang J. (1985). Preliminary observations on the embryonic development of *Hynobius leechii*. J. Dalian Med. Univ.

[81] Cai M.Z., Zhang J., Lin D.J. (1985). Preliminary observations on the embryonic development of Chinese Hynobiidae. J. Herpetol..

